# Knowledge, Attitudes, and Practices Regarding Skin Care in Saudi Arabia: A Cross-Sectional, Survey-Based Study

**DOI:** 10.7759/cureus.32490

**Published:** 2022-12-13

**Authors:** Sahar H Alsharif, Shumukh H Alqahtani, Rahaf M Alqarehi, Mayar A Alsayed, Amjd S Alzahrani, Abdullah M Alharthi, Asma S Alruwaili, Mohammed A AlFada

**Affiliations:** 1 Department of Dermatology, King Saud University, Riyadh, SAU; 2 College of Medicine, Umm Al-Qura University, Makkah, SAU; 3 College of Medicine, Northern Border University, Arar, SAU

**Keywords:** skin care, saudi arabia, practices, attitudes, knowledge, behaviors, skincare

## Abstract

Background: Skin care is the maintenance of the hygienic state of the skin toward optimal conditions of cleanliness and comfort and includes skin cleansing, skin product use, and photoprotection.

Aim: This study aimed to assess skincare knowledge, attitudes, and practices among Saudi populations.

Methods: This cross-sectional study was conducted in Saudi Arabia from February to April 2022. Data were collected using a survey distributed through social media to participants of both genders aged 12 years and above.

Results: A total of 710 subjects participated in this study. More than half were aged between 18 and 30 years (54.8%), had a bachelor’s degree (56.2%), had no income (55.8%), and resided in the Western region (58.0%). Females represented most respondents (77.3%). Skin disease was prevalent among 19.9% of the population. The combined skin type was most frequently reported for the face (52.1%), while for the body, the dry skin type predominated (42.8%). Using skincare products was significantly associated with the female gender (p < 0.001).

Conclusions: The knowledge and practices concerning of skin care are acceptable within the Saudi population. Being a female and having oily facial skin influenced skincare behavior the most among the Saudi population.

## Introduction

Skin care involves the maintenance of the hygienic state of the skin toward optimal conditions of cleanliness and comfort. Therefore, it is the range of practices that support skin integrity, enhance its appearance, and relieve any adverse symptoms, particularly regarding the skin of the face, as this reflects the health of the body. Skin care is a routine procedure performed daily in many settings, dependent on whether the skin is either too dry or too moist. Cosmetic products are generally synthetic or natural compounds or mixtures of both that contain a wide range of chemicals to which we are exposed every day, including skincare ointments, lotions, and powders [1-4].

In Saudi Arabia, income does not influence attitudes toward cosmetic products. Specifically, women in Saudi Arabia spend an average of Saudi riyal (SR) 14,256 on cosmetics yearly, representing the highest percentage in the Gulf Cooperation Council (42%) [5]. A study conducted in France by Ficheux et al. analyzing the consumption patterns of 106 products found that adult women used an average of 27 cosmetic products per year compared to 12 products used by adult men [6]. Sabharwal et al. conducted a study regarding the usage pattern and brand saliency of cosmetics in women, especially facial skin care. This study showed that moisturizers were the most common cosmetic products used among all age groups [7]. Some evidence suggests that the tangible quality of cosmetic products impacts their purchase. Furthermore, consumers prefer to purchase famous brand names [4].

Many studies have investigated the use patterns, influential factors, and basic skincare practices among women across different age intervals and economic statuses. A study at Najran University by Amar focused on women’s knowledge and attitudes toward cosmetics usage, their behavior toward cosmetics, and its effect on their budget. The authors concluded that women’s educational level greatly impacts their purchasing behavior in response to advertisements. Most women read products’ ingredient lists before purchase, indicating that most are well-informed regarding what their skin is being exposed to and whether these are harmful to them. Some do not examine the product to determine whether it is approved by the Ministry of Health in its country of origin [4].

A study conducted in Thailand concluded that gender and education level significantly influence knowledge of and practice in skin care among adolescents. They stated that most adolescents knew the effects of sunlight, but only a minority of them applied sunscreen regularly. Persons, social media, print media, and television/radio accounted for 88.5%, 77.5%, 30.7%, and 26.1%, respectively, of their sources of knowledge. Female teenagers use moisturizers, cosmetics, and sunscreen significantly more than males, while high school-aged teenagers use cosmetics more than those in junior high school [8].

Yinuo in his study explains that women buy cosmetics either because of the brand, price, friends’ recommendations, or the packaging design [9]. Since this area remains largely unexplored among the Saudi population, this study aimed to assess skincare knowledge, attitudes, and practices among Saudis to fill this knowledge gap.

## Materials and methods

Study design and population

This cross-sectional, survey-based study was conducted between February and April 2022. The survey was distributed electronically via social media to men and women in all regions of Saudi Arabia aged 12 years and above.

This study was approved by the institutional review board (IRB) of Umm Al-Qura University's Research Ethics Committee (approval number: HAPO-02-K-012-2022-03-1036).

Study procedure and questionnaire structure

An online questionnaire was designed using Google Forms (Google, Mountain View, CA) and distributed electronically via social media platforms for approximately one month. All data were entered automatically into an Excel sheet (Microsoft Corporation, Redmond, WA) and then into RStudio statistical programming software (RStudio, Boston, MA) for analysis. The questionnaire was composed of three sections with a total of 30 questions. The first section covered the sociodemographic information of the participants, which included age, gender, education level, pre-existing skin diseases, and personal monthly monetary allowance. The second section evaluated knowledge about, attitudes toward, and practices in skin care. Questions in this section focused on the benefits of cleansing, cleansing frequency, cleansing duration, the effect of moisturizer, reasons for skin product use, appropriate timing of moisturizer application, skincare products currently being used, effects of sunlight, sun protection practices, proper sun protection factor (SPF), and where to apply sunscreen. The third section inquired about the sources of skincare information and which of these they felt they could trust.

Statistical analysis

Statistical analyses were performed in RStudio (R version 4.1.1). Frequencies and percentages were used to present the variables. When applicable, the factors associated with using skin products were assessed using univariate tests, including Pearson’s chi-squared test or Fisher’s exact test. The independently associated factors were further investigated by incorporating the significant elements in a multivariate binary logistic regression analysis. Results were presented as odds ratios (ORs) with their respective 95% confidence intervals (95% CIs). Statistical significance was considered at p < 0.05.

## Results

Sociodemographic characteristics

In the current study, we analyzed the responses of 710 participants. More than half of the respondents were aged between 18 and 30 years (54.8%), had a bachelor’s degree (56.2%), had no income (55.8%), and were residing in the Western region (58.0%). Females represented most respondents (77.3%). Skin disease was prevalent among 19.9% of the sample. Regarding skin type, the most common type of face skin was the combined type (52.1%), whereas the most frequently reported skin type of the body was the dry type (42.8%, Table 1).

**Table 1:** Sociodemographic characteristics * The variable has five missing values.

Parameter	Category	N (%)
Region	Western	412 (58.0%)
	Central	101 (14.2%)
	Eastern	37 (5.2%)
	Northern	25 (3.5%)
	Southern	135 (19.0%)
Age*	<18	50 (7.1%)
	18 to <30	386 (54.8%)
	30 to <45	174 (24.7%)
	45 to <60	91 (12.9%)
	60 or more	4 (0.6%)
Gender	Male	161 (22.7%)
	Female	549 (77.3%)
Education	None	6 (0.8%)
	Primary	2 (0.3%)
	Middle school	24 (3.4%)
	Secondary	197 (27.7%)
	Diploma	56 (7.9%)
	Bachelor	399 (56.2%)
	Postgraduate	26 (3.7%)
Income (Saudi riyal)	No income	396 (55.8%)
	<3,000	0 (0.0%)
	3,000 to 5,000	101 (14.2%)
	5,000 to 8,000	80 (11.3%)
	>10,000	133 (18.7%)
Suffer from dermal diseases	Yes	141 (19.9%)
Face skin type	Do not know	76 (10.7%)
	Oily	176 (24.8%)
	Normal	0 (0.0%)
	Dry	88 (12.4%)
	Combination	370 (52.1%)
Body skin type	Do not know	153 (21.5%)
	Oily	59 (8.3%)
	Normal	0 (0.0%)
	Dry	304 (42.8%)
	Combination	194 (27.3%)

Characteristics of body and face cleaning

More than half of the respondents (59.9%) said they washed their faces more than three times daily. Based on participants’ responses, the most commonly perceived benefits of taking a shower were getting rid of body smell (90.0%), skin cleaning (89.9%), and getting rid of sweat (81.4%). Half of the respondents indicated that they take a shower more than three times daily, and the shower duration was more than 20 minutes among 30.7% of the sample. Most participants (69.2%) preferred warm water while taking a shower (Table 2).

**Table 2:** Characteristics of body and face cleaning * An item with multiple response answers.

Parameter	Category	N (%)
Benefits of taking a shower*	Clean the skin	638 (89.9%)
	Get rid of micro-organisms	550 (77.5%)
	Get rid of sweat	578 (81.4%)
	Get rid of body smell	639 (90.0%)
	Moisturize the skin	233 (32.8%)
	Skin whitening	196 (27.6%)
	No useful	1 (0.1%)
	Do not know	3 (0.4%)
Frequency of taking a shower per week	Once	14 (2.0%)
	Twice	83 (11.7%)
	Three times	258 (36.3%)
	More than three times	355 (50.0%)
Frequency of washing the face per day	Once	73 (10.3%)
	Twice	146 (20.6%)
	Three times	66 (9.3%)
	More than three times	425 (59.9%)
Shower duration	<3 minutes	4 (0.6%)
	5 to 10 minutes	120 (16.9%)
	10 to 15 minutes	177 (24.9%)
	15 to 20 minutes	191 (26.9%)
	>20 minutes	218 (30.7%)
Shower water temperature	Do not know	8 (1.1%)
	Room temperature	57 (8.0%)
	Warm water	491 (69.2%)
	Cold water	27 (3.8%)
	Hot water	127 (17.9%)

The perceived benefits and patterns of using the skin products

The most frequently reported benefits of skin products included skin moisturization (82.0%), skin cleansing (79.4%), and reducing skin rashes (55.2%). About two-thirds of the sample under study indicated that they used skin products (68.3%, Table 3).

**Table 3:** The perceived benefits and patterns of using the skin products

Parameter	Category	N (%)
Benefits of skin products	Clean the skin	564 (79.4%)
	Moisturize the skin	582 (82.0%)
	Reduce rashes	392 (55.2%)
	Reduce oily secretions	361 (50.8%)
	Skin whitening	370 (52.1%)
	Wash out the sweat	156 (22.0%)
	Has a cosmetic effect	373 (52.5%)
	Reduce dark circles	268 (37.7%)
	Reduce scars	197 (27.7%)
	Not useful	11 (1.5%)
	Do not know	35 (4.9%)
Use of skin products	Yes	485 (68.3%)
	No	225 (31.7%)

Using skin products was significantly associated with the female gender (74.7% among females vs. 46.6% among males, p < 0.001). Additionally, skin product use increased with age in which higher proportions of participants aged between 30 and 45 years (73.0%) and between 45 and 60 years (73.6%) indicated the use of skin products compared to those aged <18 years (60.0%) and 18 to <30 years (66.8%, p = 0.012). There were also significant differences based on the face skin types, in which the use of skin products was significantly higher among participants with a combined skin type (75.9%) and the dry type (64.8%) compared to those with the oily type (58.0%, Table 4).

**Table 4:** Factors associated with using the skin products

Parameter	Category	Using skin products		
		No, N = 225	Yes, N = 485	P-value
Region	Western	132 (32.0%)	280 (68.0%)	0.533
	Central	28 (27.7%)	73 (72.3%)	
	Eastern	16 (43.2%)	21 (56.8%)	
	Northern	8 (32.0%)	17 (68.0%)	
	Southern	41 (30.4%)	94 (69.6%)	
Age	<18	20 (40.0%)	30 (60.0%)	0.012
	18 to <30	128 (33.2%)	258 (66.8%)	
	30 to <45	47 (27.0%)	127 (73.0%)	
	45 to <60	24 (26.4%)	67 (73.6%)	
	60 or more	4 (100.0%)	0 (0.0%)	
Gender	Male	86 (53.4%)	75 (46.6%)	<0.001
	Female	139 (25.3%)	410 (74.7%)	
Education	None	4 (66.7%)	2 (33.3%)	0.181
	Primary	1 (50.0%)	1 (50.0%)	
	Middle school	10 (41.7%)	14 (58.3%)	
	Secondary	70 (35.5%)	127 (64.5%)	
	Diploma	18 (32.1%)	38 (67.9%)	
	Bachelor	116 (29.1%)	283 (70.9%)	
	Postgraduate	6 (23.1%)	20 (76.9%)	
Income	None	124 (31.3%)	272 (68.7%)	0.518
	<3,000	0 (NA)	0 (NA)	
	3,000 to 5,000	37 (36.6%)	64 (63.4%)	
	5,000 to 8,000	21 (26.2%)	59 (73.8%)	
	>10,000	43 (32.3%)	90 (67.7%)	
Suffer from dermal diseases	Yes	44 (31.2%)	97 (68.8%)	0.890
Face skin type	Do not know	31 (40.8%)	45 (59.2%)	<0.001
	Oily	74 (42.0%)	102 (58.0%)	
	Normal	0 (NA)	0 (NA)	
	Dry	31 (35.2%)	57 (64.8%)	
	Combination	89 (24.1%)	281 (75.9%)	
Body skin type	Do not know	59 (38.6%)	94 (61.4%)	0.174
	Oily	18 (30.5%)	41 (69.5%)	
	Normal	0 (NA)	0 (NA)	
	Dry	86 (28.3%)	218 (71.7%)	
	Combination	62 (32.0%)	132 (68.0%)	

To identify any independently associated variables, we constructed a multivariate logistic regression model with the significantly associated variables from the univariate analysis. Results showed that only gender was an independent predictor of using skin products, where females will be more likely to use skin products (OR = 3.16, 2.08 to 4.82, p < 0.001, Table 5).

**Table 5:** Predictors of skin product use * The records are not available because the variable has one arm with a zero value.

Parameter	Category	OR	95% CI	P-value
Age	<18			
	18 to <30	1.89	0.99, 3.55	0.050
	30 to <45	1.79	0.90, 3.52	0.093
	45 to <60	1.97	0.91, 4.28	0.084
	60 or more	NA*	NA	0.971
Gender	Male			
	Female	3.16	2.08, 4.82	<0.001
Face skin type	Do not know			
	Oily	0.9	0.50, 1.60	0.724
	Dry	0.78	0.39, 1.55	0.481
	Combination	1.4	0.78, 2.46	0.252

Characteristics of using skin products

**Figure 1:**
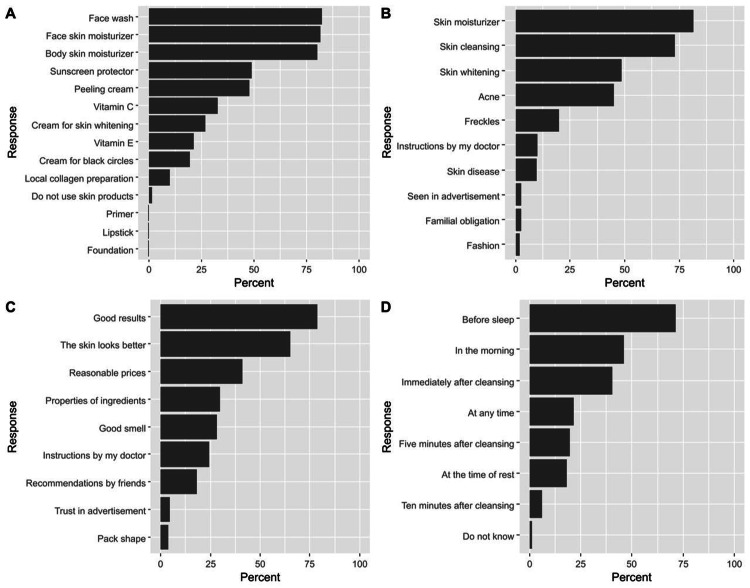
Participants’ responses regarding the characteristics of using skin products, including the types of skin products (A), causes of using the products (B), causes of selecting the products (C), and the best time of applying a moisturizing cream (D) The responses were retrieved from participants who indicated the use of skin products (n = 485).

Focusing on skin product users (n = 485), the most commonly used types of skin products included facial cleansers (82.3%), face lotion (81.4%), and body lotion (80.0%, Figure 1A). The most common reasons for using these products were skin moisturization (81.6%), skin cleansing (73.2%), and skin whitening (48.4%, Figure 1B). Most respondents selected skin products because they achieved good results (78.7%) and their skin looked better with product application (65.2%, Figure 1C). The best times for product application were before sleep (71.3%), in the morning (46.2%), and immediately after cleaning (40.2%, Figure 1D).

Participants’ perceptions regarding the effects of the sun on the skin and the utility of sunscreen

Most participants indicated that skin darkening and sunburns were the most common undesirable effects of the sun on the skin (85.4% and 60.4%, respectively) and that it is necessary to use sun protection preparations (86.2%). The most commonly perceived means of protecting against the undesirable effects of the sun were to apply sunscreen (86.3%), wear sunglasses (53.1%), and stay in the shade (50.7%). The most common areas to apply sunscreen were the face (78.3%), hands (67.3%), and areas exposed to the sun (52.4%, Table 6).

**Table 6:** Participants’ perceptions regarding the effects of the sun on the skin and the utility of sunscreen preparations * Items with multiple response answers.

Parameter	Category	N (%)
Effects of the sun on skin*	Skin darkening	606 (85.4%)
	Skin burn	429 (60.4%)
	Vitamin synthesis	252 (35.5%)
	Skin cancer	204 (28.7%)
	Freckles	255 (35.9%)
	Early aging	232 (32.7%)
	Body health	141 (19.9%)
	Bone strength	161 (22.7%)
	No effect	2 (0.3%)
	Do not know	45 (6.3%)
The necessity of sun protection	Do not know	61 (8.6%)
	Not necessary	37 (5.2%)
	Necessary	612 (86.2%)
The right method for sun protection*	Apply sunscreen protectors	613 (86.3%)
	Stay in darkness	360 (50.7%)
	Using umbrellas	183 (25.8%)
	Wearing protective clothes	153 (21.5%)
	Wearing hats	169 (23.8%)
	Wearing sunglasses	377 (53.1%)
Suitable sun protection factor for daily use	Do not know	321 (45.2%)
	<15	73 (10.3%)
	15 to 30	85 (12.0%)
	30 to 50	153 (21.5%)
	>50	78 (11.0%)
Suitable areas to apply sunscreen*	Face	556 (78.3%)
	Hands	478 (67.3%)
	Legs	93 (13.1%)
	Neck	329 (46.3%)
	Chest and abdomen	50 (7.0%)
	Entire body	49 (6.9%)
	Areas exposed to the sun	372 (52.4%)
	Lips	75 (10.6%)
	Do not know	71 (10.0%)

The practice of using sunscreen

**Figure 2:**
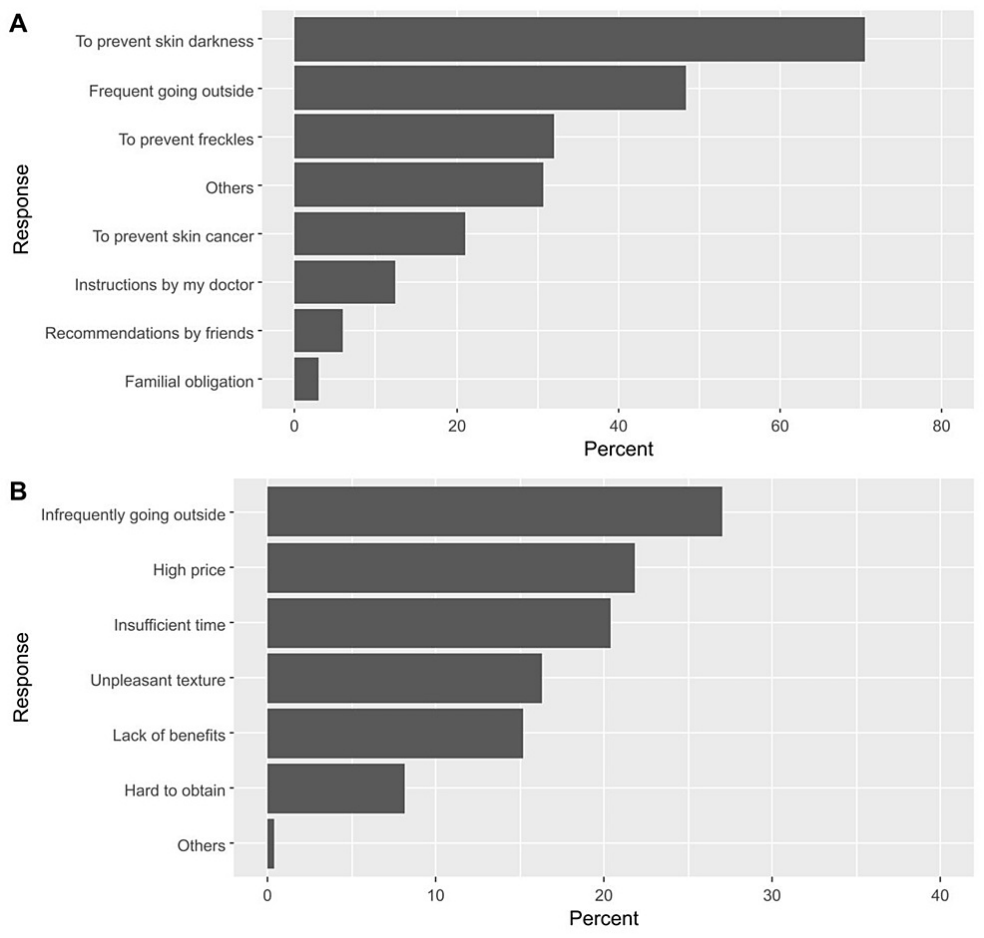
Participants’ responses regarding the causes of using sunscreen protectors (A) and not using sunscreen protectors (B)

Among the participants, 270 respondents (38.0%) indicated that they did not use sunscreen, while around half of the participants used sunscreen irregularly, and 120 used them regularly (every two hours while exposed to the sun, 16.9%). Among the users, the most common reasons for using sunscreen were to prevent skin darkness (70.5%), to spend time outdoors (48.4%), and avoid developing freckles (32.0%, Figure 2A). Focusing on non-users, the most frequently reported reasons for not using sunscreen were going outside infrequently (27.3%), high price (21.9%), and not having time to apply sunscreen before going outdoors (20.4%, Figure 2B).

Sources of information about skin care

**Figure 3:**
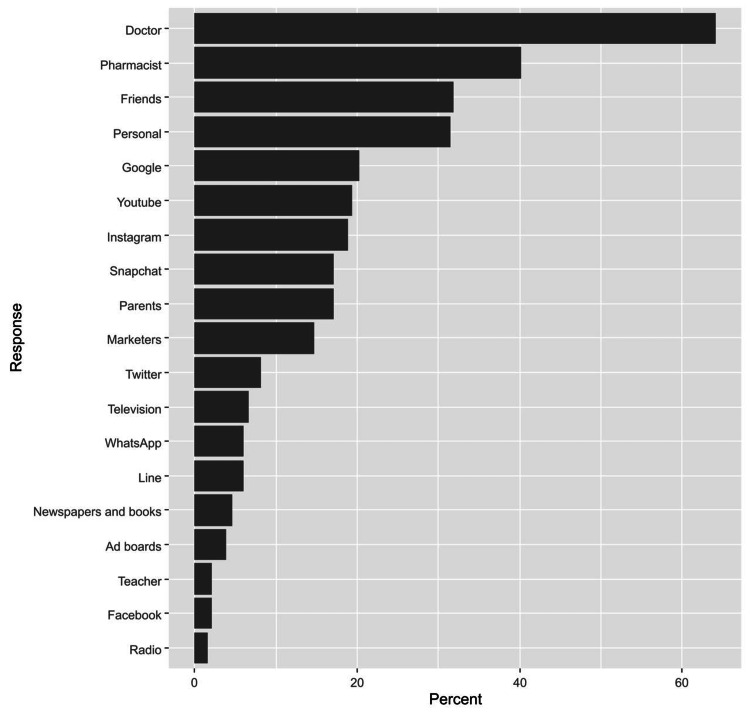
Participants’ responses regarding the sources of information about skin care

Physicians were the most common source of information regarding skincare products (64.1%). These were followed by pharmacists (40.1%), friends (31.8%), and personal information (31.4%, Figure 3).

## Discussion

The skin is the largest organ in the human body. It is frequently exposed to different physical and chemical substances daily [10]. Skincare practices are vital in keeping and endorsing healthy skin conditions. Frequent skincare practices include cleansing, moisturizer application, and photoprotection [11,12]. Many factors, including stress, inflammations, medication, and chemicals, may affect the skin’s barrier function, namely, its ability to maintain integrity [13]. Consequently, optimizing the person's general healthy practices can help to sustain or protect normal skin integrity.

The current study aimed to assess skincare knowledge, attitudes, and practices among Saudi people. More than half of the respondents had a combined facial skin type, while less than half had a body dry skin type. Only one-fifth of the study participants suffered from skin disease. It also showed that the vast majority of the study participants knew that showering cleans skin and removes body smell, sweat, and microorganisms. Regarding shower practice, nearly half of the respondents reported showering more than three times a week, and about two-thirds washed their faces more than three times per day. Showering for more than 30 minutes was reported by less than one-third of the respondents and two-thirds used warm water to shower. The hot climate may impact these behaviors.

In most countries, people do not shampoo their hair every time they shower. On the contrary, Mexicans and Japanese people come closest to fully sanitizing their hair each time [14]. A literature review revealed that only 7% of Brazilians take a bath, and 99% report taking weekly showers. On average, Brazilians take two showers a day or 14 showers a week while 83% of the UK population report taking weekly showers. In Germany, 92% of residents take showers, whereas only 20% choose to take baths. While 85% of Chinese people prefer using showers over bathtubs to bathe, only 11% do so. Showering is undoubtedly the most convenient and economical way to bathe, especially given China's acknowledged population difficulties [15].

The current study revealed that more than two-thirds of the study participants used skincare products. The most reported benefits, according to our study participants, were moisturizing, cleaning, and reducing rashes. Higher skin product utilization was associated with increased age and the female gender due to cosmetic issues. Similar results were obtained by Alrayyes et al. [16], who reported that 65% of Saudis use topical skin-lightening products, and 32.7% had previously used these products. A study by Al-Aojan et al. [17] found that topical corticosteroids (TCS) were used by 43.1% of respondents. Being female was the only identified risk factor for using TCS without a prescription. The most reported reasons for using TCS were for treating pruritus without skin disease (23.7%) and cosmetic reasons (19.9%). In Nigeria, Egbi et al. [18] found that 40.9% of the females used skin products, with facial cleansers being the most common product used (51.1%). The removal of discoloration/dark spots (40%) and "cosmetic reasons" (37.8%) were the most reported reasons for the use of skincare products. More than 80% of participants knew skin-lightening products could cause adverse effects, with "skin irritation" being the most known (64.5%).

Concerning participants’ perception of the effects of the sun on the skin, our study showed that most of the participants believed that skin darkening and sunburn were the most common harmful effects of the sun on the skin and that it is necessary to use sun protection. The most commonly perceived methods for protection against the undesirable effects of the sun were to apply sun protectors, wear sunglasses, and stay in the shade. The most common areas to apply sunscreen were the face (78.3%), hands (67.3%), and areas exposed to the sun. In Saudi Arabia, another study showed concordant results where 94.8% agreed that sun exposure could cause sunburns, and the majority knew the association between sun exposure and skin cancer. About two-thirds of the participants in that study knew about the association between sun exposure and skin aging and hyperpigmentation. Also, more than 80% of participants knew that the period of exposure to sunlight that causes the most harm is between 10:00 am and 2:00 pm [19]. Also, Agarwal et al. [20] found that the majority of their participants were aware that sun exposure could cause darkening, but awareness of the other effects of sun exposure, such as wrinkling, melasma, allergy, and skin cancer, was less common. About 68% of their upper-class respondents knew about sunscreen, while most of the respondents from the lower-middle class did not know about sunscreens.

The present study has some limitations. Because no descriptions of the skin types were included in the survey, some participants might have misjudged their skin type. Another limitation is that the electronic questionnaire was distributed on social media, which reduces the credibility of the participants' answers. Moreover, the number of females who answered the questionnaire exceeded the number of males, which may lead to a gender bias.

## Conclusions

This study offers baseline data on skincare knowledge, attitudes, and practices among the Saudi population across a range of several sociodemographic strata. The female gender and oily skin type of the face were the two significant factors that affect skincare behavior in our study. Although knowledge and practice of skin care are at a good level according to our study. However, an accurate and comprehensive awareness campaign seems necessary to promote the level of this knowledge and practices.
